# Supplementary material for: CDKN2A Homozygous Deletion Is a Stronger Predictor of Outcome than IDH1/2-Mutation in CNS WHO Grade 4 Gliomas
Source: Biomedicines. 2024 Oct 4;12(10):2256. doi: 10.3390/biomedicines12102256 (PMC11505494; doi:10.3390/biomedicines12102256)
Supplement: Supplementary file 1 [file biomedicines-12-02256-s001.zip › Supplementary Figures S1-S5.pdf]

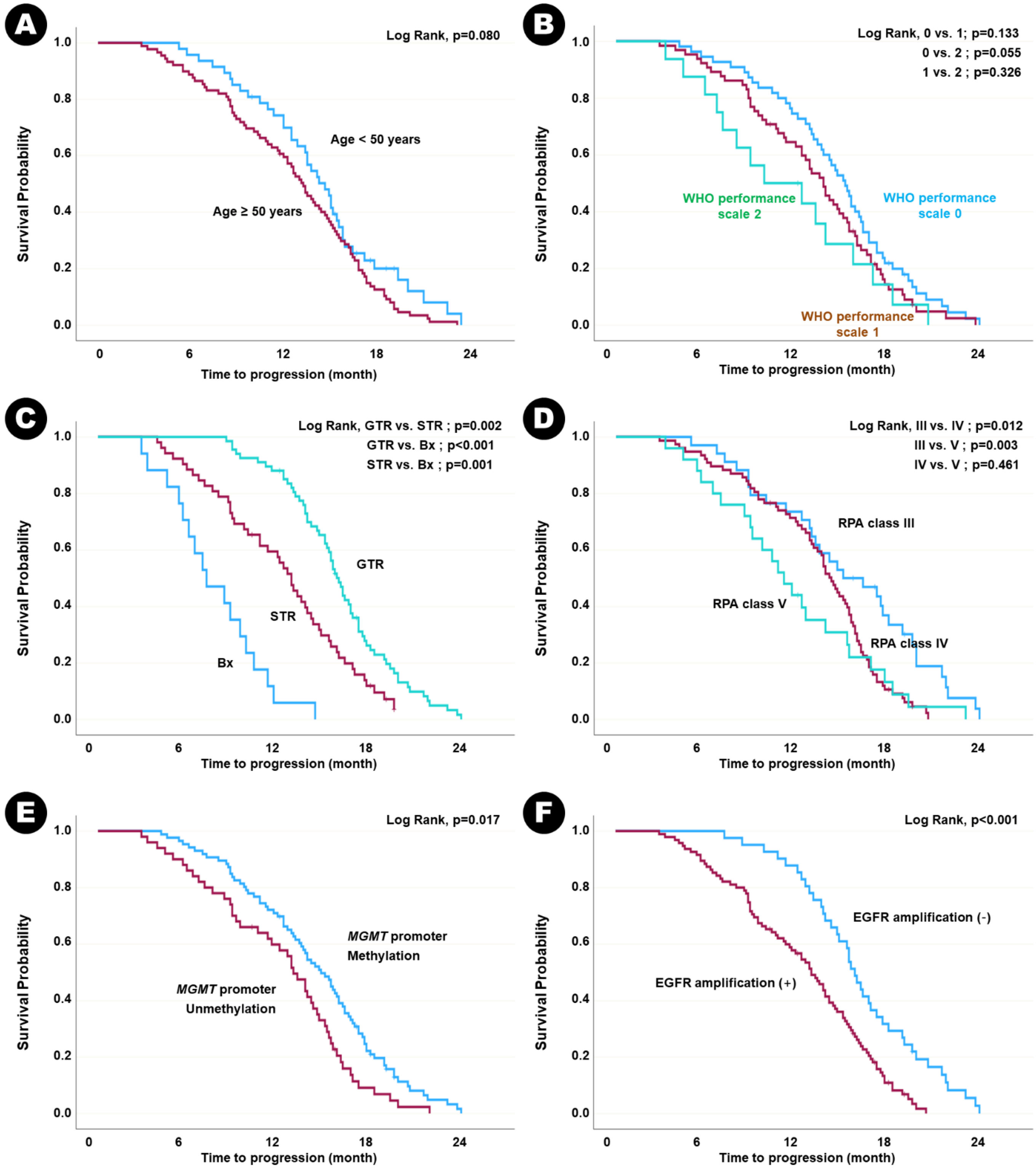
A
Log Rank, p=0.080
Age < 50 years
Age ≥ 50 years
Survival Probability
Time to progression (month)
B
Log Rank, 0 vs. 1; p=0.133
0 vs. 2 ; p=0.055
1 vs. 2 ; p=0.326
WHO performance scale 2
WHO performance scale 0
WHO performance scale 1
Survival Probability
Time to progression (month)
C
Log Rank, GTR vs. STR ; p=0.002
GTR vs. Bx ; p<0.001
STR vs. Bx ; p=0.001
GTR
STR
Bx
Survival Probability
Time to progression (month)
D
Log Rank, III vs. IV ; p=0.012
III vs. V ; p=0.003
IV vs. V ; p=0.461
RPA class III
RPA class V
RPA class IV
Survival Probability
Time to progression (month)
E
Log Rank, p=0.017
MGMT promoter Methylation
MGMT promoter Unmethylation
Survival Probability
Time to progression (month)
F
Log Rank, p<0.001
EGFR amplification (-)
EGFR amplification (+)
Survival Probability
Time to progression (month)

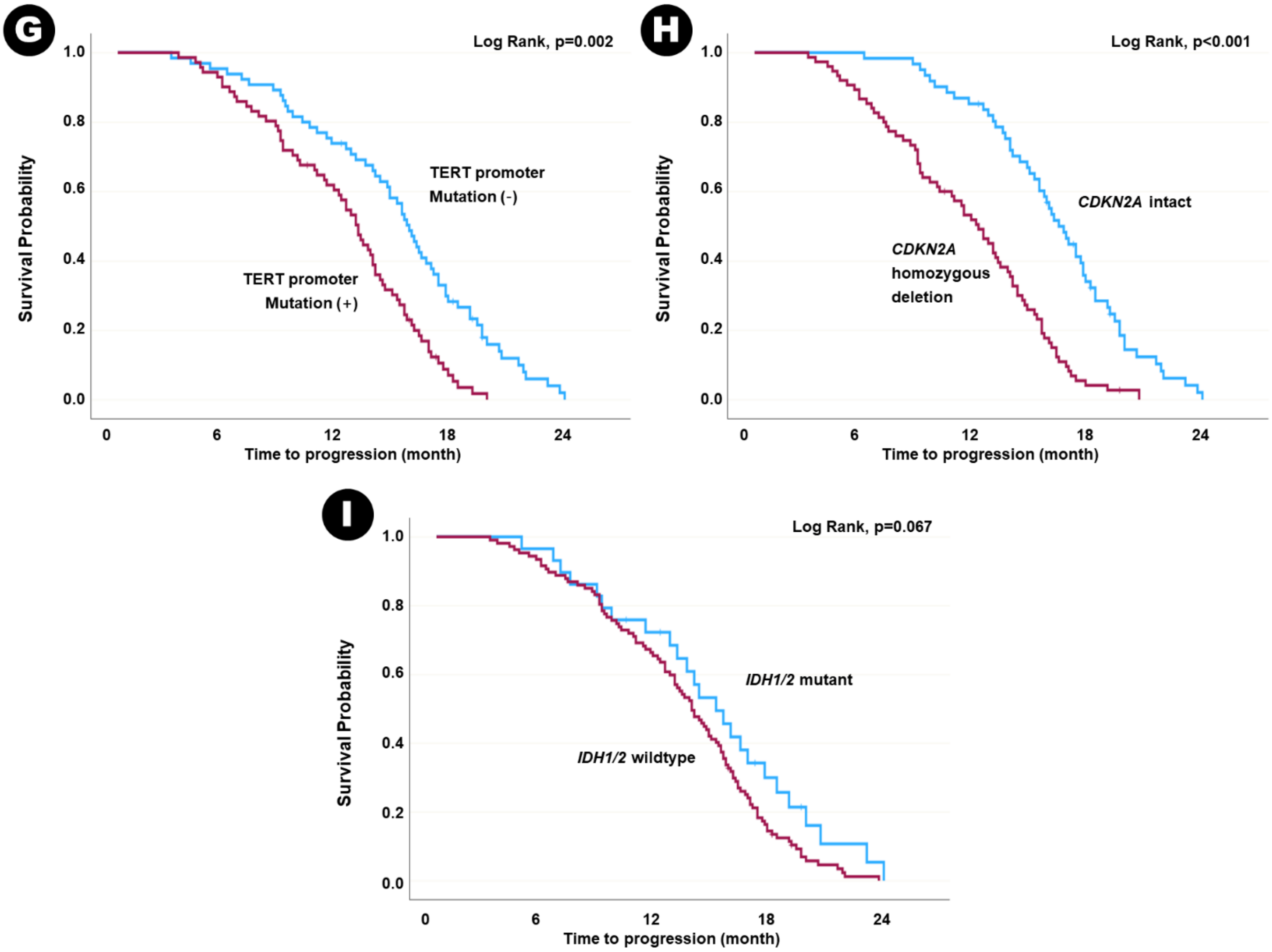

Figure S1. Kaplan-Meier survival curve shows that following factors are associated with progression-free survivals; extent of resection (C), RPA class (D), *MGMT* gene promoter methylation (E), EGFR amplification (F), *TERT* promoter mutation (G), and *CDKN2A* homozygous deletion (H). However, age (A), WHO performance status (B), and *IDH1/2* mutation (I) are not statistically associated with progression-free survival. Abbreviations**.** *CDKN2A*, cyclin-dependent kinase inhibitor 2A; EGFR, epidermal growth factor receptor; *IDH*, isocitrate dehydrogenase; *MGMT,* O6-methyl guanine DNA methyltransferase; RPA, recursive partitioning analysis; *TERT,* telomerase reverse transcriptase, WHO, World Health Organization.

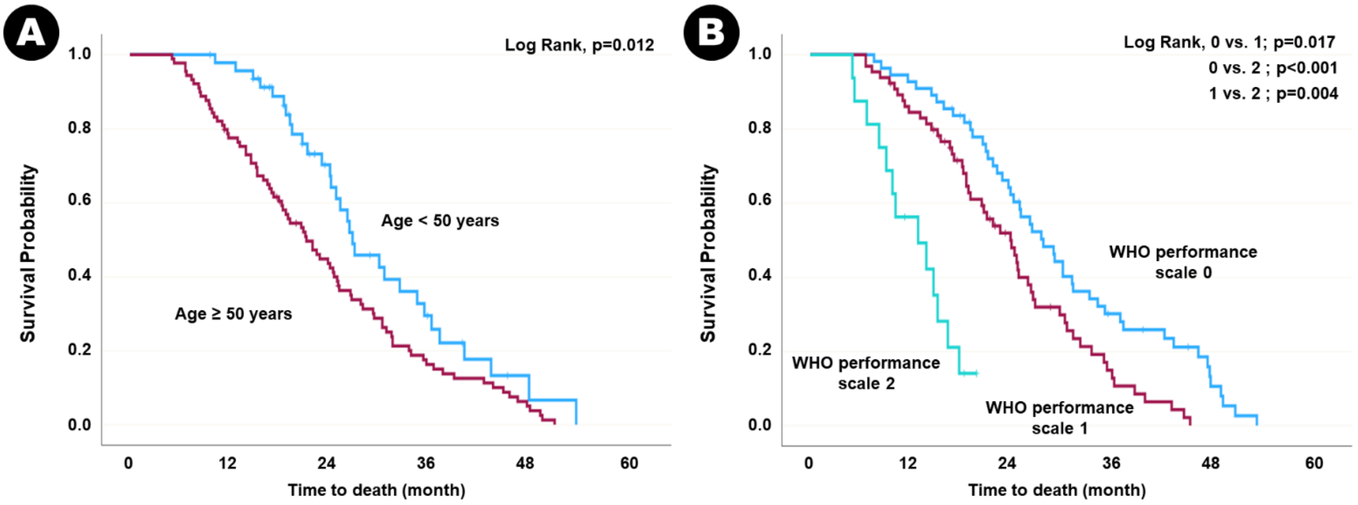

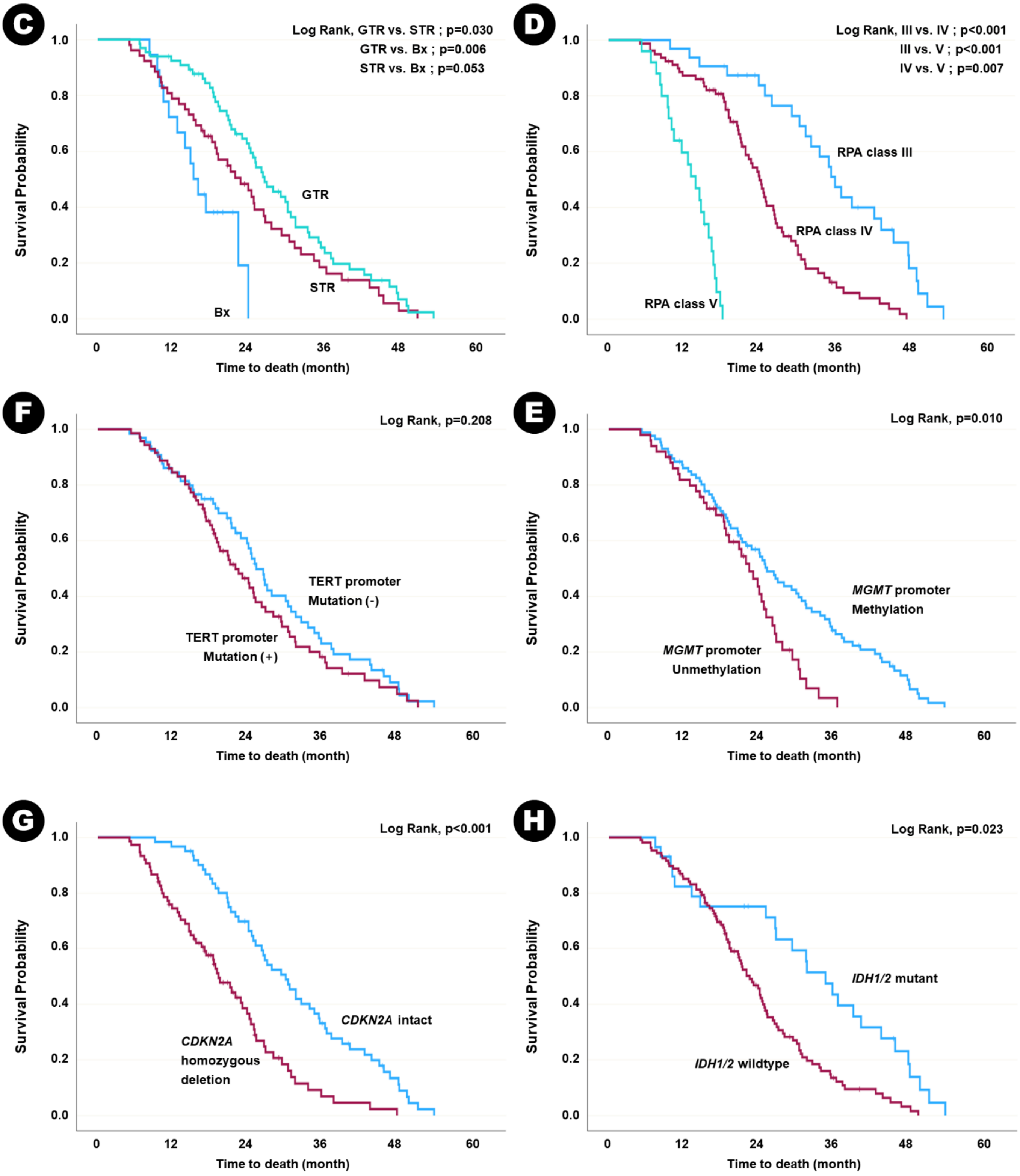
C
Log Rank, GTR vs. STR ; p=0.030
GTR vs. Bx ; p=0.006
STR vs. Bx ; p=0.053
Survival Probability
1.0
0.8
0.6
0.4
0.2
0.0
GTR
STR
Bx
0
12
24
36
48
60
Time to death (month)
D
Log Rank, III vs. IV ; p<0.001
III vs. V ; p<0.001
IV vs. V ; p=0.007
Survival Probability
RPA class III
RPA class IV
RPA class V
Time to death (month)
F
Log Rank, p=0.208
Survival Probability
TERT promoter
Mutation (-)
TERT promoter
Mutation (+)
Time to death (month)
E
Log Rank, p=0.010
Survival Probability
MGMT promoter
Methylation
MGMT promoter
Unmethylation
Time to death (month)
G
Log Rank, p<0.001
Survival Probability
CDKN2A intact
CDKN2A
homozygous
deletion
Time to death (month)
H
Log Rank, p=0.023
Survival Probability
IDH1/2 mutant
IDH1/2 wildtype
Time to death (month)

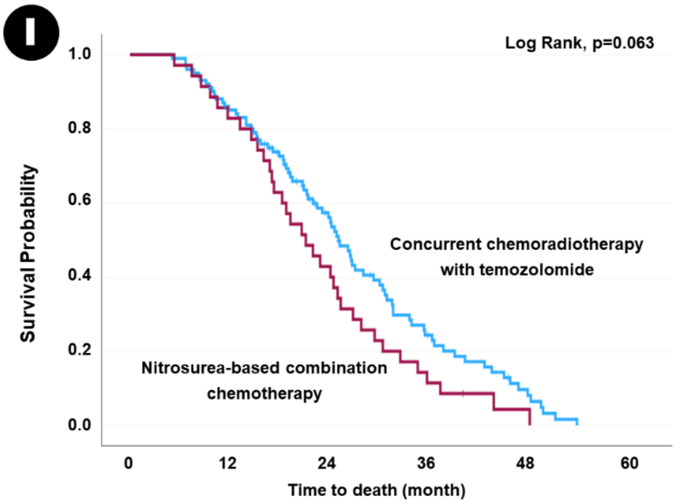

Figure S2. Kaplan-Meier survival curve shows that following factors are associated with overall survivals; age (A), WHO performance status (B), extent of resection (C), RPA class (D), *MGMT* gene promoter methylation (E), *CDKN2A* homozygous deletion (G) and *IDH1/2* mutation (H). However, *TERT* promoter mutation (F), and postoperative therapeutic modality (I) are not statistically associated with overall survival. Abbreviations. *CDKN2A*, cyclin-dependent kinase inhibitor 2A; *IDH*, isocitrate dehydrogenase; *MGMT*, O6-methyl guanine DNA methyltransferase; RPA, recursive partitioning analysis; *TERT*, telomerase reverse transcriptase; WHO, World Health Organization.

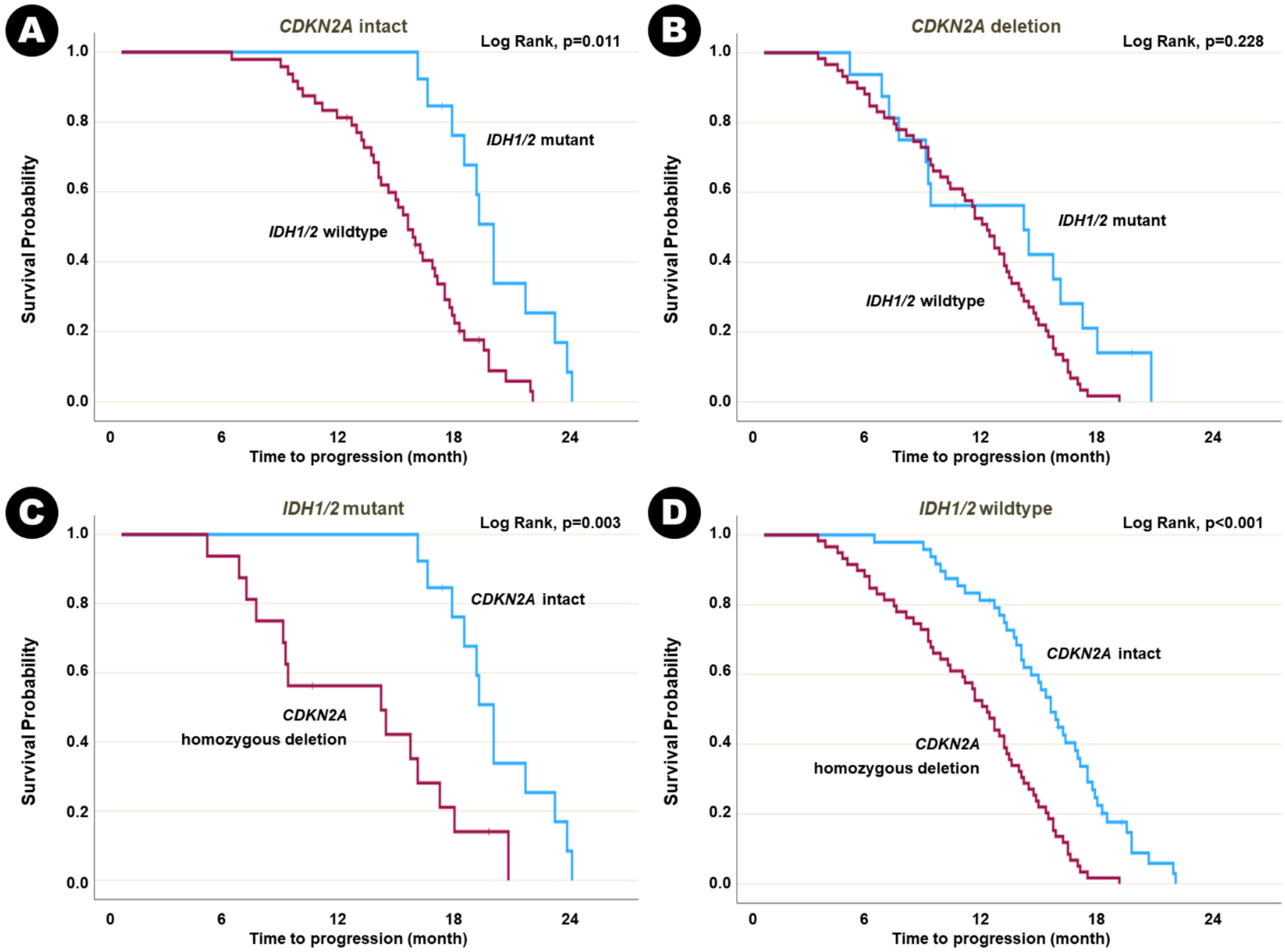

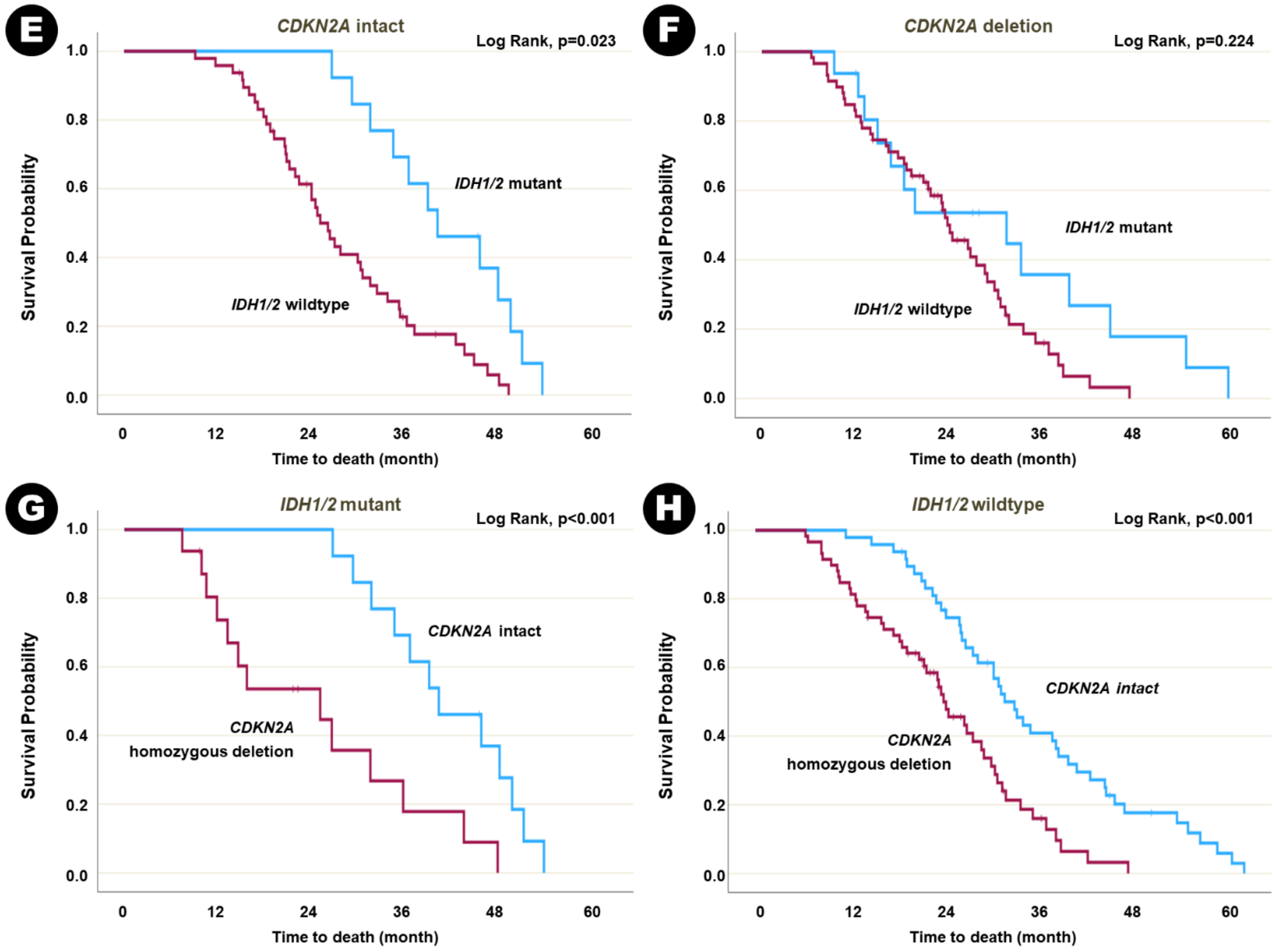

Figure S3. Combined role of *CDKN2A* homozygous deletion and *IDH1/2* mutation on progression-free survival (A-D) and overall survival (E-H) in patients with CNS WHO grade 4 gliomas. *CDKN2A* homozygous deletion is associated with short progression-free survival (C and D) and overall survival (G and H) with or without *IDH1/2* mutation, but *IDH1/2* mutation did not influence on progression-free survival (B) and overall survival (F) with *CDKN2A* homozygous deletion. Abbreviations**.** *CDKN2A*, cyclin-dependent kinase inhibitor 2A; CNS, central nervous system; *IDH*, isocitrate dehydrogenase; WHO, World Health Organization.

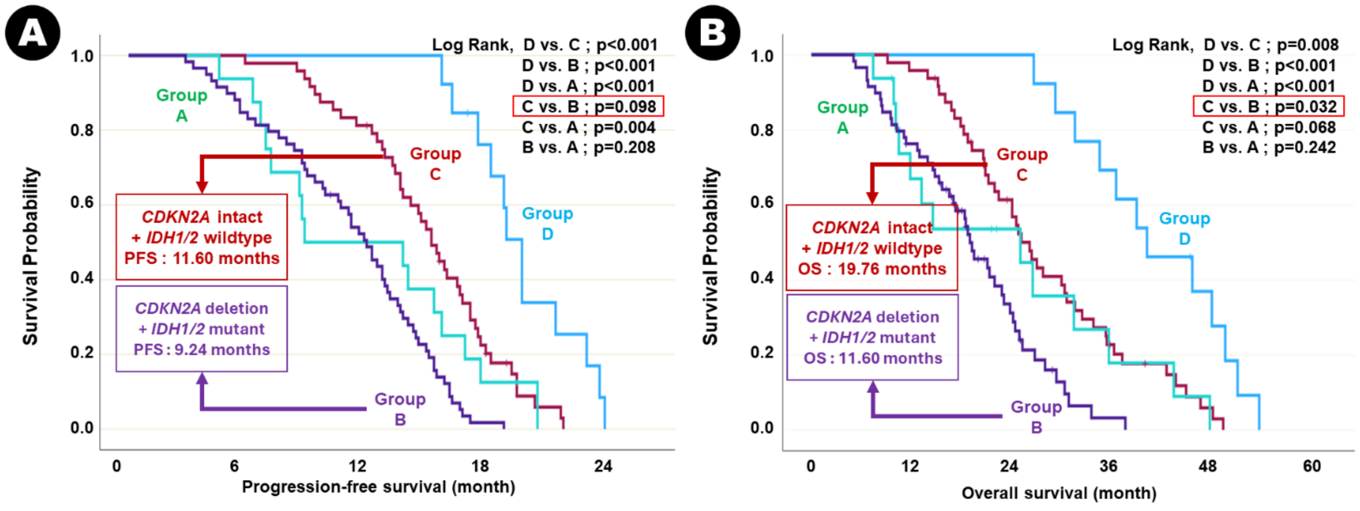

Figure S4. Progression-free survival (PFS) and overall survival (OS) according to the *CDKN2A* deletion and *IDH1/2* mutation) in patients with CNS WHO grade 4 gliomas. In this subgroup analysis, even patients with

IDH1/2 mutation (group B) have shorter PFS and OS than patients with *IDH1/2* wildtype if they are accompanied by *CDKN2A* deletion (group C). Abbreviations. *CDKN2A*, cyclin-dependent kinase inhibitor 2A; CNS, central nervous system; *IDH*, isocitrate dehydrogenase; WHO, World Health Organization.

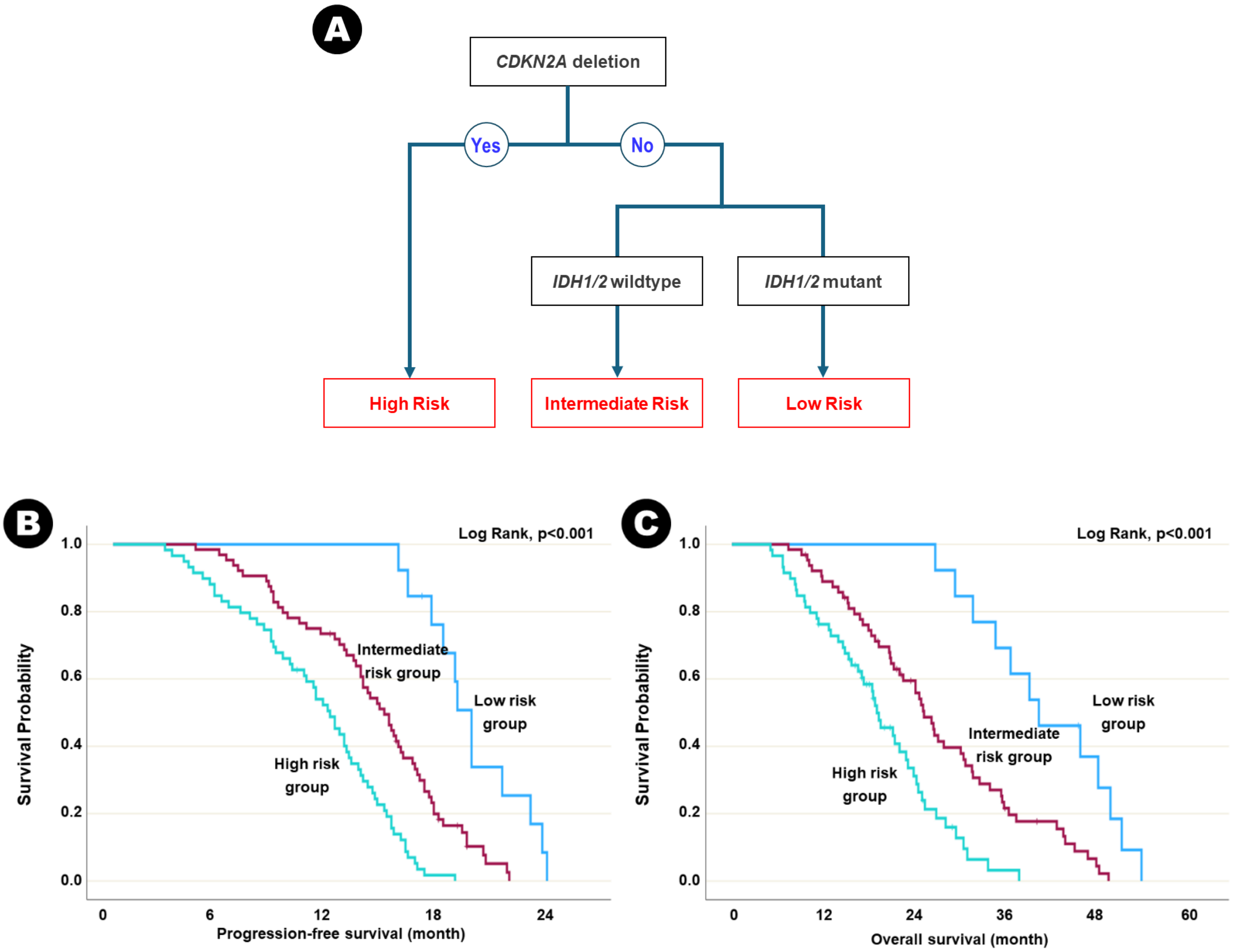

Figure S5. Progression-free survival (PFS) and overall survival (OS) according to the risk groups in patients with CNS WHO grade 4 gliomas. High risk group has *CDKN2A* homozygous deletion regardless *IDH1/2*-mutation, intermediate risk group has intact *CDKN2A* with *IDH1/2*-mutation, and low risk group has intact *CDKN2A* with *IDH1/2*-mutation (A). Kaplan-Meier's curve shows the significantly different PFS (B) and OS (C) among each group. Abbreviations. *CDKN2A*, cyclin-dependent kinase inhibitor 2A; *IDH*, isocitrate dehydrogenase; WHO, World Health Organization.
