# Supplementary material for: CDKN2A Homozygous Deletion Is a Stronger Predictor of Outcome than IDH1/2-Mutation in CNS WHO Grade 4 Gliomas
Source: Biomedicines. 2024 Oct 4;12(10):2256. doi: 10.3390/biomedicines12102256 (PMC11505494; doi:10.3390/biomedicines12102256)
Supplement: Supplementary file 1 [file biomedicines-12-02256-s001.zip › Supplementary Tables S2-S5 (20240927).pdf]

Table S2. Univariate analysis of factors predicting **progression-free survival (PFS)** in WHO grade 4 glioma cohorts using Cox regression model focused on *CDKN2A* deletion and *IDH* mutation.

	Mean PFS (month, ±SD)	Hazard Ratio	95% CI	p-value
**Group A**<br>: *CDKN2A* deletion + *IDH* wildtype	8.53 (±0.694)			
**Group B**<br>: *CDKN2A* deletion + *IDH* mutant	9.72 (±0.725)	1.586	0.861-2.311	0.208
**Group C**<br>: *CDKN2A* intact + *IDH* wildtype	11.60 (±0.816)	2.740	0.915-4.565	0.097
**Group D**<br>: *CDKN2A* intact + *IDH* mutant	15.25 (±1.124)	11.979	8.879-15.079	**<0.001**

Abbreviation. *CDKN2A*, cyclin-dependent kinase inhibitor 2A; CI, confidence interval; *IDH*, isocitrate dehydrogenase; PFS, progression-free survival.

Table S3. Univariate analysis of factors predicting **overall survival (OS)** in WHO grade 4 glioma cohorts using Cox regression model focused on *CDKN2A* deletion and *IDH* mutation.

	Mean OS (month, ±SD)	Hazard Ratio	95% CI	p-value
**Group A**<br>: *CDKN2A* deletion + *IDH* wildtype	15.63 (±1.302)			
**Group B**<br>: *CDKN2A* deletion + *IDH* mutant	19.67 (±1.525)	1.872	0.887-20857	0.240
**Group C**<br>: *CDKN2A* intact + *IDH* wildtype	22.63 (±2.009)	3.043	0.989-5.097	0.058
**Group D**<br>: *CDKN2A* intact + *IDH* mutant	33.38 (±2.536)	12.455	9.627-15.283	**<0.001**

Abbreviation. *CDKN2A*, cyclin-dependent kinase inhibitor 2A; CI, confidence interval; *IDH*, isocitrate dehydrogenase; OS, overall survival.

Table S4. Mean **progression-free survival (PFS)** according to CDKN2A deletion status in WHO grade 4 glioma cohorts.

		CDKN2A deletion (n=75 / ±SD)	CDKN2A intact (n=61 / ±SD)
Age (years)	<50	9.86 (±0.721)	12.45 (±0.995)
	≥50	8.32 (±0.629)	15.98 (±1.233)
Sex	Male	8.64 (±0.600)	11.89 (±0.898)
	Female	8.93 (±0.655)	13.19 (±1.107)
WHO performance status score	0	9.62 (±0.705)	13.59 (±1.223)
	1	8.49 (±0.693)	12.09 (±0.946)
	2	7.16 (±0.628)	11.08 (±0.869)
Extent of surgery	Gross total resection	9.85 (±0.751)	15.02 (±1.365)
	Subtotal resection	8.72 (±0.702)	10.26 (±0.924)
	Biopsy	4.89 (±0.322)	7.46 (±0.555)
RPA class	III	10.31 (±0.795)	13.23 (±0.989)
	IV	9.00 (±0.711)	11.74 (±0.864)
	V	6.51 (±0.432)	10.56 (±0.722)
MGMT gene promoter	methylated	9.40 (±0.785)	12.97 (±1.117)
	unmethylated	7.66 (±0.528)	11.39 (±0.799)
EGFR amplification	No	9.42 (±0.709)	16.77 (±1.235)
	Yes	8.43 (±0.669)	10.71 (±0.935)
TERT promoter mutation	No	9.64 (±0.967)	13.25 (±1.212)
	Yes	8.12 (±0.638)	11.33 (±0.825)
IDH mutation	Yes	9.72 (±0.705)	15.25 (±1.351)
	No	8.53 (±0.687)	11.60 (±0.952)
Postoperative adjuvant therapy			
RTx and/or Nitrosourea chemotherapy		8.70 (±0.688)	11.79 (±0.995)
CCRT with Temozolomide		8.76 (±0.680)	12.70 (±1.023)

Abbreviation. CCRT, Concurrent chemoradiotherapy; *CDKN2A*, cyclin-dependent kinase inhibitor 2A; CI, confidence Interval; EGFR, epidermal growth factor receptor; *IDH*, isocitrate dehydrogenase; GTR,

Gross Total Resection; *MGMT*, O6-methyl DNA guanine methyltransferase; RPA, recursive partitioning analysis; RTx, radiotherapy; STR, Subtotal Resection; *TERT*, telomerase reverse transcriptase; WHO, World Health Organization.

Table S5. Mean **overall survival (OS)** according to CDKN2A deletion status in WHO grade 4 glioma cohorts.

		CDKN2A deletion (n=75 / ±SD)	CDKN2A intact (n=61 / ±SD)
Age (years)	<50	21.52 (±2.525)	26.67 (±3.054)
	≥50	14.76 (±1.351)	25.32 (±2.721)
Sex	Male	16.02 (±1.624)	25.40 (±2.785)
	Female	17.71 (±1.708)	24.44 (±2.700)
WHO performance status score	0	20.22 (±2.285)	28.14 (±3.235)
	1	16.22 (±1.768)	23.63 (±2.264)
	2	7.84 (±0.635)	14.29 (±1.325)
Extent of surgery	Gross total resection	18.67 (±1.720)	28.31 (±3.008)
	Subtotal resection	15.93 (±1.324)	24.05 (±2.852)
	Biopsy	11.47 (±0.906)	16.60 (±1.729)
RPA class	III	22.05 (±2.009)	36.67 (±4.631)
	IV	16.70 (±1.391)	24.05 (±2.205)
	V	9.52 (±0.858)	11.11 (±0.879)
MGMT gene promoter	methylated	19.12 (±1.994)	26.09 (±2.885)
	unmethylated	12.50 (±1.062)	23.40 (±2.725)
EGFR amplification	No	17.76 (±1.694)	28.07 (±3.226)
	Yes	16.13 (±1.505)	24.85 (±2.168)
TERT promoter mutation	No	17.74 (±1.721)	25.63 (±2.533)
	Yes	15.88 (±1.448)	25.82 (±2.605)
IDH mutation	Yes	19.67 (±2.253)	33.38 (±3.542)
	No	15.63 (±1.113)	22.63 (±2.084)
Postoperative adjuvant therapy			
RTx and/or Nitrosourea chemotherapy		16.81 (±1.733)	19.81 (±1.956)
CCRT with Temozolomide		16.60 (±1.659)	28.73 (±2.792)

Abbreviation. CCRT, Concurrent chemoradiotherapy; *CDKN2A*, cyclin-dependent kinase inhibitor 2A; CI, confidence Interval; EGFR, epidermal growth factor receptor; *IDH*, isocitrate dehydrogenase; GTR, Gross Total Resection; *MGMT*, O6-methyl DNA guanine methyltransferase; RPA, recursive partitioning analysis; RTx, radiotherapy; STR, Subtotal Resection; *TERT*, telomerase reverse transcriptase; WHO, World Health Organization.
